# MACULAR ATROPHY FINDINGS BY OPTICAL COHERENCE TOMOGRAPHY ANGIOGRAPHY COMPARED WITH FUNDUS AUTOFLUORESCENCE IN TREATED EXUDATIVE AGE-RELATED MACULAR DEGENERATION

**DOI:** 10.1097/IAE.0000000000001980

**Published:** 2017-11-28

**Authors:** Yukari Takasago, Chieko Shiragami, Mamoru Kobayashi, Rie Osaka, Aoi Ono, Ayana Yamashita, Akitaka Tsujikawa, Kazuyuki Hirooka

**Affiliations:** * Department of Ophthalmology, Kagawa University Faculty of Medicine, Kagawa, Japan; and; † Department of Ophthalmology and Visual Sciences, Kyoto University Faculty of Medicine, Kyoto, Japan.

**Keywords:** macular atrophy, choriocapillaris nonperfusion, fundus autofluorescence, optical coherence tomography angiography, exudative age-related macular degeneration, choroidal ischemia

## Abstract

**Purpose:**

To compare the areas of choriocapillaris (CC) nonperfusion and macular atrophy (MA) in treated exudative age-related macular degeneration.

**Methods:**

This was a prospective, observational, cross-sectional study. Forty-four eyes exhibiting MA (42 patients with age-related macular degeneration), with a dry macula, underwent fundus autofluorescence and optical coherence tomography angiography. The area of MA detected by fundus autofluorescence and CC nonperfusion detected by optical coherence tomography angiography was measured using image analysis software. The rates of concordance between the MA and CC nonperfusion areas were calculated. We qualitatively and quantitatively compared the areas of MA and CC nonperfusion in age-related macular degeneration eyes.

**Results:**

The mean areas of MA and CC nonperfusion were 5.95 ± 4.50 mm^2^ and 10.66 ± 7.05 mm^2^, respectively (paired *t*-test, *P* < 0.001). In 39 eyes (88.6%), the CC nonperfusion area was larger than the MA area, and the mean CC nonperfusion area was significantly larger than the mean MA area. Fundus autofluorescence matching optical coherence tomography angiography showed that the CC nonperfusion area was almost included in the MA area. The mean concordance rate for the MA area inside the CC nonperfusion area was 87.7 ± 13.9%.

**Conclusion:**

The MA and CC nonperfusion areas markedly overlapped. The area of CC nonperfusion correlated with the MA area. Choroidal ischemia might be involved in the pathogenesis of MA in treated age-related macular degeneration.

## Abstract 2

The area of choriocapillaris nonperfusion on optical coherence tomography angiography correlated with the area of macular atrophy on fundus autofluorescence in eyes with exudative age-related macular degeneration. Choroidal ischemia might be involved in macular atrophy in treated age-related macular degeneration.

Exudative age-related macular degeneration (AMD) is a leading cause of vision loss in elderly people globally. Loss of vision from this disease is mostly due to the development of neovascular AMD and development of retinal pigment epithelium (RPE) atrophy. Results from the comparison of AMD treatments trials, in which patients were treated for 2 years with anti–vascular endothelial growth factor (VEGF) agents, showed that the 2-year incidence of geographic atrophy (GA) was approximately 18%.^1^ When GA was present at the fovea, the visual acuity was markedly decreased.^2,3^ Grunwald et al^2^ described a number of risk factors associated with faster GA growth in patients with AMD treated with anti-VEGF medications for a period of 2 years.

In general, color fundus photography and fundus autofluorescence (FAF) have been used to measure the lesion of GA in eyes with atrophic and exudative AMD.^4,5^ Because FAF imaging allows for investigation of RPE function, reduced FAF signals may correspond to the area of RPE atrophy or loss. Consequently, GA may indicate severely reduced FAF signals at the area of RPE atrophy and fibrotic scars. Geographic atrophy has previously been defined as RPE atrophy as observed in atrophic AMD in numerous studies.^4–6^ Recently, the term “macular atrophy (MA)” has been commonly substituted for GA in end-stage exudative AMD.^6,7^ Indeed, MA caused by RPE atrophy and fibrotic scars is the late-stage, atrophic manifestation of AMD. The appearance of MA is an important indicator of poor visual prognosis in eyes with AMD.^8^ Although MA causes loss of central vision after treatment of exudative lesions, its pathogenesis has remained unknown.

Given that the maintenance of the RPE and outer retina is important for good vision,^9^ the choroid is also considered to play an important role in visual acuity. The choroidal circulation provides nutrients to and removes metabolic wastes from the RPE and outer retina.^10,11^ Therefore, an impaired choroidal circulation likely disrupts normal retinal function, leading to visual deterioration. It has been well documented that choroidal flow and choriocapillaris (CC) volume are negatively correlated with aging.^12^

In AMD eyes, vascular changes have been visualized using angiographic examinations. However, these angiography procedures are invasive and cause several allergic events. Optical coherence tomography angiography (OCTA) is a relatively new imaging technique that generates three-dimensional images of vasculature in vivo, without dye injection. It can be used to visualize the state of chorioretinal blood flow in each layer, noninvasively. In previous reports, hyporeflectivities beneath GA may represent nonperfused or hypoperfused choroidal vessels with nondetectable flow in patients with GA secondary to atrophic AMD.^13,14^ However, there have been no reports to date on the use of OCTA for examining MA in eyes with exudative AMD.

In this study, we compared the CC nonperfusion area as measured by OCTA, and the MA area as measured by FAF, and the relationship was evaluated.

## Methods

This prospective, observational, cross-sectional study was conducted between July 15, 2015, and December 6, 2015, in the Department of Ophthalmology of Kagawa University Hospital. Written informed consent was obtained from each participant before any of the study procedures or examinations were performed, and the study was approved by the Ethics Committee at Kagawa University Faculty of Medicine (Kagawa, Japan) and was conducted in accordance with the tenets of the Declaration of Helsinki.

### Patient Selection and Examination

We enrolled patients with exudative AMD who had developed a completely dry macula and presented with MA after treatment with intravitreal anti-VEGF agents and/or photodynamic therapy (PDT). The inclusion criteria were as follows: 1) age >65 years; 2) diagnosis of exudative AMD, with a completely dry macula; 3) attainment of dry macula at least 6 months before inclusion in the study; 4) MA shown on FAF, which indicated a severely reduced FAF signals area of RPE atrophy and fibrotic scars involving fovea; and 5) CC nonperfusion area indicated clearly demarcated on OCTA. The exclusion criteria included the following: 1) signs of exudative changes, including subretinal fluid, pigment epithelial detachment, and hemorrhage, 2) presence of any other retinal disorder potentially confounding the clinical assessment (e.g., diabetic retinopathy and retinal vein occlusion), 3) indistinct MA without sharp outlines, 4) myopia greater than 6 diopters, 5) any previous treatment with direct laser photocoagulation, and 6) presence of significant media opacities. Cases in which MA and CC nonperfusion areas were larger than 8.7 × 8.7 mm and 8 × 8 mm, respectively, were also excluded.

Each patient underwent comprehensive ophthalmologic examinations, including best-corrected visual acuity, color photography, OCT, FAF, and OCTA, on the same day.

### Measurement of Macular Atrophy, Increased Fundus Autofluorescence, and Choriocapillaris Nonperfusion Area

Macular atrophy was identified on color photographs as RPE atrophy, and on FAF as regions with absent autofluorescence. Macular atrophy was defined as RPE atrophy, including fibrotic scars, after treatment. Fundus autofluorescence examinations were performed using the Spectralis (Heidelberg Engineering, Heidelberg, Germany). Using image analysis software (Heidelberg Engineering, Version 6.3), which was built into the device, MA areas were measured manually. The boundary line was almost clear, and a concentric area of MA, including the fovea, was selected for measuring the size of the MA areas.

Choi et al reported that low decorrelation signals may be due to the complete absence of flow and vasculature, which is considered atrophy on OCTA images.^15^ Therefore, we defined “CC nonperfusion” as hyporeflectivity at the choroidal capillary level. To measure the area of CC nonperfusion, the instrument used for the OCTA images was based on the RTVue XR Avanti (Optovue, Inc, Fremont, CA). En face OCTA images were also used to assess the presence of a choroidal flow void signal surrounding the vascular lesion, as hyporeflective areas at the level of the CC. The hyporeflectivity at the choroidal capillary level was measured as a reduction or loss of an area of CC perfusion using image analysis software built into the device (Optovue, Version 2016.1.0.26). To ensure correct visualization and assessment of the CC layer, we used the automatic segmentation provided by the system software, with minor manual adjustments. If a hyperreflective suprachoroidal vessel or choroidal neovascularization was observed within the CC nonperfusion area on OCTA, it was defined as the area of CC nonperfusion.

### Comparison of Fundus Autofluorescence and Optical Coherence Tomography Angiography

The area of severely reduced FAF signals indicating MA and the area of CC nonperfusion on OCTA were compared. The images used for the examination were 8.7 × 8.7 mm for FAF and 8 × 8 mm for OCTA. All FAF and OCTA images were superimposed based on the orientation of the retinal vessels, using Photoshop CS6 (Adobe Systems Inc, San Jose, CA) image editing software. All measurements were performed independently by two raters (Y.T. and C.S.). In cases where there was a difference greater than 15% between the measurements obtained by the 2 observers, arbitration through open adjudication was performed. In the few cases in which agreement was not achieved, a resolution was established by a third expert grader on evaluation of the images (A.T.). An average of the measurements of the two observers was used for statistical analysis.

Using ImageJ image-processing package (http://rsb.info.nih.gov/ij/), the FAF and OCTA images were superimposed using retinal vessels as a landmark, and the areas of MA and CC nonperfusion were measured in pixels. The rate of concordance in each area was calculated.

### Statistical Analyses

Statistical analysis was performed with SPSS software version 21.0 (IBM SPSS Statistics, Chicago, IL). For comparing the MA area and CC nonperfusion area, paired *t*-tests and Pearson correlation were used. The mean matching rate between the MA area and CC nonperfusion area was compared with AMD phenotype (one-way analysis of variance, with post hoc Tukey test). Unpaired *t*-tests were used to compare the MA area and CC nonperfusion area between eyes treated with combined PDT and those treated with anti-VEGF therapy alone without PDT. Pearson correlation coefficients were used to investigate the correlation between the total number of injections and CC nonperfusion area. *P* values <0.05 were considered statistically significant.

## Results

This study enrolled 44 eyes of 42 patients with exudative AMD in which a completely dry macula had developed, and which showed MA after treatment with intravitreal anti-VEGF agents alone (31 eyes) and combined PDT (13 eyes). Patient baseline characteristics are summarized in Table 1.

**Table 1.**
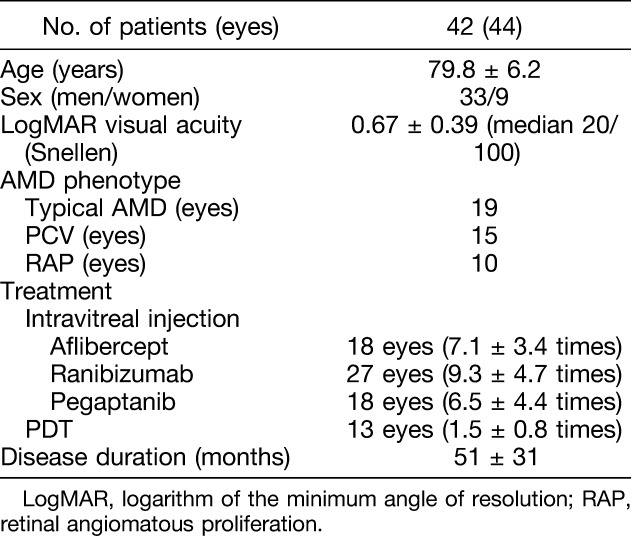
Clinical Characteristics of Patients With MA in AMD

Most CC nonperfusion areas measured on OCTA were larger than the MA area measured on FAF. The mean matching rate between the MA area and CC nonperfusion area was 87.7 ± 13.9%. There were no significant differences in concordance rate between typical AMD and polypoidal choroidal vasculopathy (PCV) (*P* = 0.890), between typical AMD and retinal angiomatous proliferation (*P* = 0.975), or between PCV and retinal angiomatous proliferation (*P* = 0.955).

**Fig. 1.**
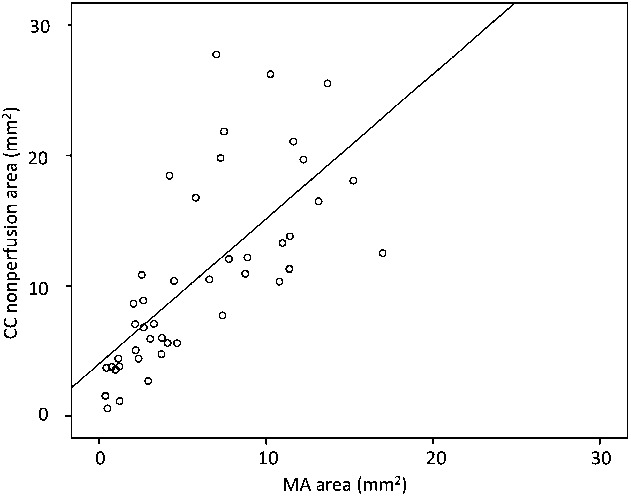
Relationship between the CC nonperfusion area and MA area. There is a linear strong relationship between the two variables (r = 0.708, *P* < 0.001).

The area of CC nonperfusion in all cases included more than 50% of the MA area. The MA area was completely encompassed by the CC nonperfusion area in 11 eyes (25.0%). The mean CC nonperfusion area on OCTA was 10.66 ± 7.05 mm^2^, and the mean MA area on FAF was 5.95 ± 4.50 mm^2^. The CC nonperfusion area was significantly larger than the MA area (*P* < 0.001). The CC nonperfusion area was larger than the MA area in 39 eyes (88.6%), and there was a linear relationship between the 2 variables (r = 0.708, *P* < 0.001; Figure 1).

The mean CC nonperfusion area of eyes treated with combined PDT was 14.04 ± 6.04 mm^2^, whereas that of eyes treated with anti-VEGF therapy alone without PDT was 9.24 ± 6.96 mm^2^ (Table 2). Choriocapillaris nonperfusion areas were significantly larger in eyes treated with combined PDT than in eyes treated with anti-VEGF therapy alone (*P* < 0.05). The mean MA area of eyes treated with combined PDT was 8.42 ± 4.20 mm^2^, whereas the mean MA area of eyes treated with anti-VEGF alone was 4.91 ± 4.22 mm^2^. Macular atrophy areas were significantly larger in eyes treated with combined PDT than in eye treated with anti-VEGF therapy alone (*P* < 0.05). No significant correlation was observed between the total number of anti-VEGF injections and CC nonperfusion area (r = 0.183, *P* = 0.235).

**Table 2.**
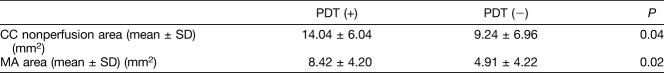
Mean CC Nonperfusion and MA Area in Patients Treated With or Without PDT

### Case Report

#### An 87-year-old man with polypoidal choroidal vasculopathy in his right eye

**Fig. 2.**
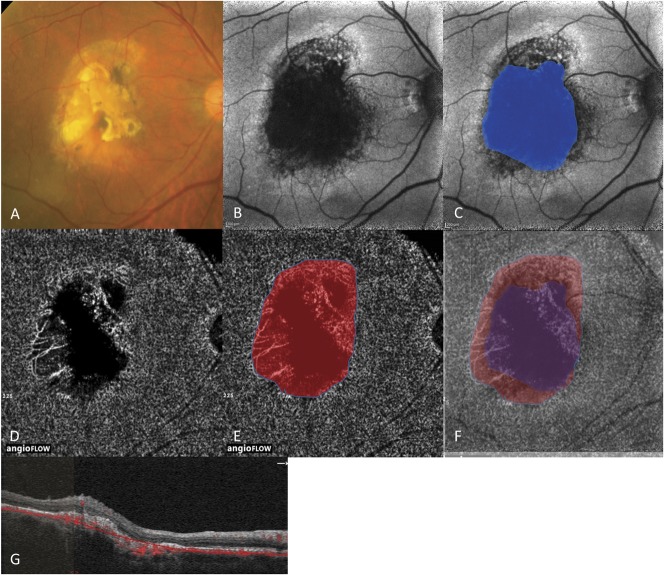
An 87-year-old man with PCV with MA in his right eye. **A.** Color fundus photograph showing a fibrous scar surrounding by an area of RPE atrophy. **B.** Fundus autofluorescence photograph showing the hypofluorescent MA lesion. **C.** Macular atrophy area (highlighted in blue) is 10.9 mm^2^. **D.** Optical coherence tomography angiography at the level of the CC showing hyporeflective CC nonperfusion. **E.** Choriocapillaris nonperfusion area (highlighted in red) is 13.2 mm^2^. **F.** Composite photograph of OCTA and FAF images showing that the MA area (highlighted in purple) was completely included in the CC nonperfusion area. **G.** Cross-sectional B scan with flow information of OCTA.

The patient had received 9 intravitreal pegaptanib injections, 11 intravitreal ranibizumab injections, and 2 PDT treatments. Color fundus photography showed a fibrotic scar surrounding an area of RPE atrophy (Figure 2A). The MA area was 10.9 mm^2^ on FAF (Figure 2B). The CC nonperfusion area was 13.2 mm^2^ on OCTA (Figure 2, D and G). A composite photograph of FAF (Figure 2C) and OCTA (Figure 2E) images showed that the MA area was completely included in the CC nonperfusion area (Figure 2F).

## Discussion

In this prospective, observation, cross-sectional study, we compared the areas of CC nonperfusion and MA in treated exudative AMD. We found a relationship between the area of MA measured on FAF, and the area of CC nonperfusion measured on OCTA, in the eyes with treated exudative AMD. Our study revealed that most CC nonperfusion areas were larger than the MA. In addition, there was a linear relationship between the CC nonperfusion areas and MA areas. These results suggest that CC nonperfusion occurs simultaneously, or earlier than MA development.

Because the maintenance of the RPE and outer retina is vital for good vision,^9^ the choroid is also considered to play an important role in visual acuity. Furthermore, choroidal circulation provides nutrients to and removes metabolic wastes from the RPE and outer retina.^10,11^ McLeod et al^16^ reported that a CC dropout was evident in the absence of RPE atrophy, resulting in a 50% decrease in the vascular area. Therefore, impaired choroidal circulation likely disrupts normal retinal function, leading to visual deterioration. It has been well documented that choroidal flow and CC volume are negatively correlated with aging.^12^ Recently, Shiragami et al^7^ investigated that topical isopropyl unoprostone (R-tech Ueno, Tokyo, Japan), which is a metabolized form of prostaglandin F2a, and a big potassium (BK, maxi-K) channel activator, may prevent MA enlargement in eyes with exudative AMD. In this study, the authors suggested that topical administration of isopropyl unoprostone may be helpful to maintain the macular function, because of increasing blood flow in the choroidal circulations.^17,18^

In previous reports, the development and enlargement of RPE atrophy in eyes treated with anti-VEGF occurred.^19,20^ The issue of VEGF-dependent ocular homeostasis has yet to be examined clinically, but preclinical data suggest that VEGF_121_ may be an essential retinal neuroprotectant during ischemic conditions.^21^ Moreover, some authors believe that pan-VEGF blockade, particularly VEGF_121_ blockade, is responsible for MA enlargement in eyes with AMD and, ultimately, poor visual prognoses.^22,23^ According to findings based on 7-year outcomes of eyes treated with ranibizumab for AMD, MA with a mean area of 9.4 mm^2^ was detected on FAF images in 98% of eyes.^23^ In another report, RPE atrophy progressed during antiangiogenic therapy of exudative AMD, and RPE atrophy developed in 61% of eyes by Month 24.^24^ VEGF_121_ signaling is involved not only in choroidal vessel formation but also in the maintenance of the CC.^25^ It is possible that the growth of CC vessels is inhibited, and accordingly, the oxygen and nutrition supply is reduced, resulting in RPE atrophy.^16^

Schmidt-Erfurth et al^26^ revealed that PDT can damage the physiologic choriocapillary layer beyond the irradiated area. Furthermore, Yamashita et al^27^ reported that 57% of eyes with PCV treated with reduced-fluence PDT exhibited mild-to-moderate nonperfusion of the CC at 1 week, although 96% exhibited recovery to pretreatment levels at 3 months. In our study, 13 of 42 patients with AMD underwent PDT before examination. We observed that CC nonperfusion and MA areas in eyes treated with PDT were significantly larger than those in eyes treated with anti-VEGF agents alone. These findings indicate that PDT treatment may influence the size of CC nonperfusion areas and lead to the development of MA. By contrast, we observed no significant correlations between the size of CC nonperfusion areas and the number of anti-VEGF treatments.

In a previous study of OCTA, pronounced CC flow impairment within the region of the GA with atrophic AMD and milder CC flow impairment extending beyond the margin of the GA was observed.^15^ In our study, the mean matching rate between the MA area and CC nonperfusion area was 87.7 ± 13.9%. Choriocapillaris nonperfusion area was significantly larger than MA area and included more than 50.0% of the MA area in all cases, whereas the MA area was fully included within the CC nonperfusion area in 25.0% of cases. The mean CC nonperfusion area was significantly larger than the MA area, and greater than the MA area in 88.6% of cases. However, the pathogenesis and the cause of expansion of RPE atrophy are not clear,^8^ MA occurred beneath or within the CC nonperfusion area; it is possible that the pathogenesis of MA may involve damage of the choroidal microvascular circulation. The outer retina is supplied with oxygen and with nutrients from the choroidal vessels. Therefore, it is possible that the influence on the outer retina secondary to circulatory disorders caused by CC nonperfusion leads to RPE atrophy. In other words, the CC might first be occluded, and MA then develops due to lack of blood flow.

In conclusion, CC nonperfusion after treatment of exudative AMD may be associated with the development of MA. The limitation of this study was its small sample size, the heterogeneity of the included patients, and use of the definitions (e.g., the areas of MA and CC nonperfusion). Future studies investigating the MA are warranted to better understand the pathogenesis and the possible prognostic value of this previously unreported finding.
